# Novel Inlay Methodology with Thermoplastic and Heating System for Durable Road Markings

**DOI:** 10.3390/polym17030361

**Published:** 2025-01-28

**Authors:** Kwan Kyu Kim, Chul Soo Jun, Hee Jun Lee, Shanelle Aira Rodrigazo, Jaeheum Yeon

**Affiliations:** 1 Korea Conformity Laboratories, Chuncheon 24341, Republic of Korea; kim89@kcl.re.kr; 2 U-TECH Co., Ltd., Chuncheon 24341, Republic of Korea; ultratechjj@gmail.com; 3 California Department of Transportation, Fresno, CA 93728, USA; heejun.lee@dot.ca.gov; 4 Department of Regional Infrastructure Engineering, Kangwon National University, Chuncheon 24341, Republic of Korea; rodrigazo.shanelleaira@kangwon.ac.kr

**Keywords:** road marking, thermoplastic polymers, inlay technique, heating system, durability

## Abstract

Road markings, such as lane dividers and pedestrian crossings, are integral in ensuring the safety of road users. However, traditional markings frequently exhibit limitations, including short lifespans, diminished visibility, and significant maintenance costs, particularly as traffic volumes increase. To address these persistent challenges, this study presents a thermoplastic road marking system that combines material innovation and advanced application techniques. Central to this approach is the portable heating system, equipped with ceramic heaters and precise temperature controls, which facilitates uniform heating while mitigating fire risks. The thermoplastic blend, processed into pre-formed sheets, was integrated with this heating technology. Together, these components enabled a two-phase process, engraving asphalt surfaces followed by sheet integration, that ensured robust adhesion and seamless bonding. Field trials conducted on various asphalt types validated the system’s reliability, demonstrating its durability under traffic loads and consistent visibility. By integrating durable materials with advanced application methods, this methodology significantly enhances the efficiency, longevity, and safety of road markings. It presents a practical and scalable solution for modern infrastructure needs. Future research will focus on evaluating the system’s long-term performance under extreme weather conditions to further optimize its applicability.

## 1. Introduction

Road infrastructure is a cornerstone of economic development, ensuring the smooth movement of goods, services, and people [1,2,3]. As global expansion intensifies, the demand for reliable transportation networks grows, making them pivotal for sustaining growth and enhancing quality of life. However, traffic safety remains a serious concern worldwide, with road accidents claiming 1.19 million lives annually [4]. In South Korea specifically, inspections have uncovered that 71% of road safety deficiencies originate from degraded or improperly installed road markings [5]. These markings are vital in reducing accidents by guiding traffic flow and delineating routes, especially in high-traffic and vulnerable areas [6,7]. Yet, traditional materials often fail because of rapid wear brought on by traffic loads and environmental exposure [8]. Inadequate or deteriorated road markings thus amplify safety risks, underscoring the need for durable and effective systems [9].

Although various materials—such as paint-based coatings, thermoplastic tapes, and MMA (Methyl Methacrylate) resins—are frequently used, they each have notable drawbacks that compromise long-term efficacy. Table 1 presents the main advantages and limitations of current road marking technologies. For example, heat-weldable tapes, widely adopted in South Korea by companies such as Daedong Safety Co. [10] and Green Factory Co. [11], offer excellent visibility and design versatility but last only about 12 months and require specialized installation. Meanwhile, pre-formed thermoplastic markings, promoted by global companies like 3M™ [12], Ennis Flint^®^ [13], and PROMAX Industries [14], feature customization, durability, and improved retroreflectivity, yet they also involve high costs, complex installation, and potential fire hazards during application. Although paint-based coatings are less expensive and simpler to apply, they deteriorate quickly under traffic and environmental stresses, resulting in frequent maintenance. Similarly, MMA resins—while durable and fast-curing—pose concerns due to hazardous chemicals, and adhesive-based markings have short service lives despite their ease of removal and strong visibility.

These limitations demonstrate a universal need for cost-effective, durable, and safer road marking solutions. Accordingly, researchers have recently concentrated on material innovations to address these gaps. Ji et al. [15], for instance, studied epoxy resins modified with thermoplastic-polyimide, noting improved tensile strength and reduced melting temperatures, both vital for repetitive loading in high-traffic zones. Nevertheless, their work was confined to specific polymeric modifiers and did not account for long-term environmental effects. Concurrently, Jo et al. [16] found that thermoplastic markings featuring lower modulus and greater deformation energy adhered more effectively to asphalt, thus emphasizing mechanical interlock. However, this finding was based on only two commercial products, restricting its scope.

Durability also depends on environmental influences and material fatigue. Kavussi et al. [17] demonstrated that polymeric sulfur-modified asphalt can enhance certain properties but may also induce brittleness, increasing delamination risks. Furthermore, Zhen et al. [18] highlighted that both mechanical interlock and chemical bonding play significant roles in asphalt-aggregate adhesion, although their study did not extend these insights to polymeric road markings. Investigating aging effects, Wang et al. [19] used molecular dynamics simulations and observed stronger adhesion in the short term for some modified asphalts, yet the long-term implications remain unclear.

In parallel, polymer modifiers such as SBS (Styrene-Butadiene-Styrene), SIS (Styrene-Isoprene-Styrene), and APAO (Amorphous Polyalphaolefin) have attracted growing attention for improving flexibility and thermal stability in thermoplastic marking systems [20,21,22]. While SBS effectively boosts viscosity and rutting resistance, studies suggest that SIS and APAO can achieve similar or superior moisture resistance and cracking performance, though their robustness across varied climatic conditions is still under investigation [20,21]. Laboratory experiments on polypropylene-based composites by Varga and Baran [22] showed promising results but require further real-world validation.

Visibility, especially in low-light or harsh weather, remains another priority for road safety. Owusu et al. [6] emphasized that retroreflectivity can diminish over time due to bead loss, chipping, and discoloration. Additionally, Zhao et al. [23] employed machine learning to predict retroreflective decay, finding that factors such as traffic volume significantly shorten the usable life of markings. Yet, these studies did not examine how tailored pigment loadings or bead concentrations might bolster both reflectivity and durability. Table 2 summarizes key studies in this area, highlighting their methodologies, findings, limitations, and the research gaps they address.

Against this background, the present study aims to develop and validate a pre-formed thermoplastic road marking technology that effectively merges the strengths of domestic and international approaches while mitigating common drawbacks like high installation costs, hazardous emissions, and rapid wear. The novelty lies in an optimized polymer formulation—comprising C5 petroleum resin, SBS, SIS, polyamide, PE (Polyethylene) wax, and select additives—to achieve a balanced combination of flexibility, adhesion, and long-term performance. Moreover, the technology adopts a two-phase heating method, featuring surface engraving followed by controlled integration of the thermoplastic sheet, thereby ensuring uniform adhesion and reducing installation errors. By incorporating additives that bolster slip resistance and enhance retroreflectivity, the proposed approach is poised to extend service life and streamline maintenance. Ultimately, this strategy addresses the immediate need for more resilient road markings while contributing to a safer and more sustainable roadway infrastructure, thereby improving overall traffic safety and economic efficiency.

## 2. Materials and Methods

This study employs a systematic approach to develop and evaluate pre-formed thermoplastic road marking materials through a series of formulation experiments and field application trials. The primary objective was to optimize the resin and powder additive ratios to achieve a balance of melting viscosity, flexibility, durability, and visual quality.

### 2.1. Materials

**Figure 1:**
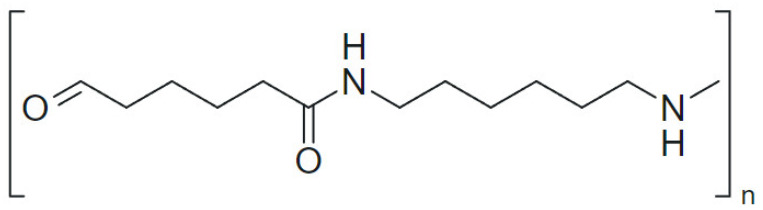
Chemical structure of Polyamide Resin with repeating amide groups (-CONH-) in the polymer backbone.

C5 Hydrogenated Petroleum Resin (HC-100, Hanwha Chemical, Seoul, South Korea) served as the primary binder, offering thermal stability, low viscosity, and excellent compatibility with other components. This resin, derived from olefin polymerization and further hydrogenated for enhanced color and odor stability, is widely used as a tackifier in thermoplastic applications [23]. Its pellet form ensures ease of processing. Polyamide Resin (SA-1135, Silverstar Chemical, Gimhae, South Korea)was added to enhance thermal resistance and mechanical strength, with its high softening point and amide group structure (-CONH-) contributing to polarity and processability [24]. The chemical structure of Polyamide, shown in Figure 1, illustrates the amide groups (-CONH-) in its polymer backbone, which enables hydrogen bonding and provides strength and flexibility.

Amorphous Poly Alpha Olefin (APAO, Hanwha Chemical, Seoul, South Korea), a copolymer of propylene, ethylene, and butene-1, acted as a plasticizer, improving adhesion and flexibility while providing oxidative stability and compatibility with the hydrogenated resin. Additionally, styrene block copolymers, including SBS, SEBS, and SIS, were explored to further enhance the material properties. These copolymers were studied for their impact on the overall flexibility, toughness, and processing characteristics of the thermoplastic road marking materials. Specifically, SBS and SEBS were investigated for their ability to improve elastic properties and thermal stability, while SIS was explored for its potential to enhance the material’s adhesion and processability. A summary of the resin material properties is outlined in Table 3. 

The powder additives, consisting of fillers, pigments, and retroreflective materials, were selected to enhance structural stability, visibility, and reflective performance. The paint used in this formulation was sourced from Jeongseok Chemical, Wangju, South Korea. Calcium Carbonate (CaCO_3_) was used as the primary filler to reduce production costs while maintaining mechanical stability and ensuring the structural integrity of the road marking material. Titanium Dioxide (TiO_2_) served as a pigment to improve whiteness and brightness, enhancing visibility under daylight conditions. Glass Beads were incorporated to provide retroreflectivity, ensuring high visibility during nighttime or low-light conditions by reflecting vehicle headlights. The specific roles and properties of these powder additives are detailed in Table 4.

### 2.2. Experimental Design

#### 2.2.1. Heating System

**Figure 2:**
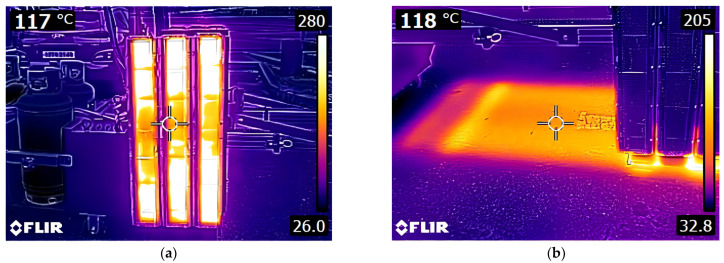
Infrared temperature measurements during heating system testing. (**a**) Heating plate temperature distribution; (**b**) Road surface heat distribution.

**Figure 3:**
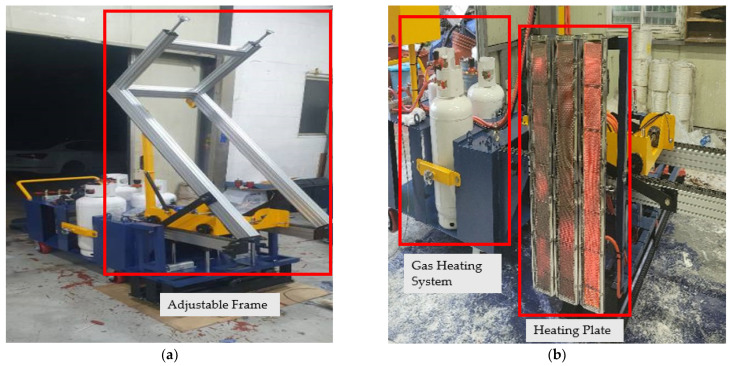
Assembly of the heating system prototype. (**a**) Frame and gas system installation; (**b**) Fully assembled prototype.

The precise application of thermoplastic road markings relied on a controlled heating process to activate polymeric materials, ensuring strong adhesion and integration with asphalt surfaces. This methodology incorporated the use of a portable gas-powered heating system, equipped with a 2.75 m × 2 m panel with adjustable height and a rail system for enhanced mobility, enabling uniform heating. Thermal imaging cameras were employed for real-time monitoring of asphalt surface temperatures to ensure they remained below 160 °C, preventing material degradation and premature aging (Figure 2). This system not only maintained optimal application conditions but also mitigated safety risks for workers by reducing direct exposure to high-temperature surfaces, ensuring a safer operational environment during installation. A rectangular ceramic heater, measuring 930 × 130 × 75 mm, was selected for its ability to deliver efficient and even heat distribution, supporting the seamless embedding of thermoplastic sheets into the asphalt (Figure 3).

#### 2.2.2. Thermoplastic Optimization

**Figure 4:**
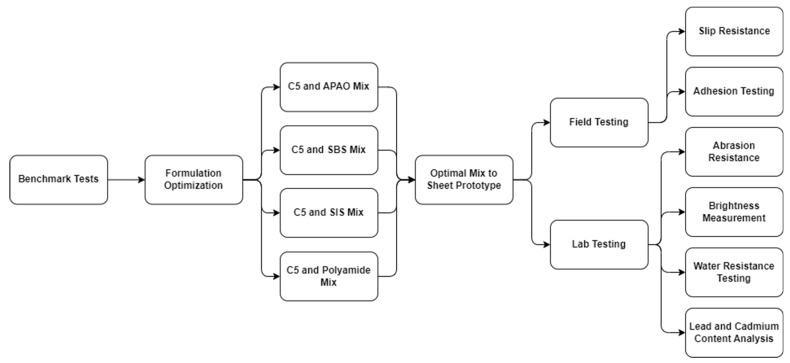
Flowchart of the thermoplastic optimization workflow.

The optimization of thermoplastic materials followed a freeform formulation approach, aimed at identifying resin and additive combinations that achieve optimal flexibility, adhesion, and durability. This adaptive process, guided by mechanical, thermal, and chemical evaluations, ensured systematic refinement. The overall experimental workflow is depicted in Figure 4, highlighting the key stages from initial benchmarking to prototype fabrication and final testing under real-world conditions.

Following Figure 4, the experimental approach proceeded in two major phases: an initial benchmarking of pure resins (and minor blends) to establish baseline behavior, followed by a comprehensive formulation optimization that systematically explored different resin–additive ratios.

The first phase, benchmarking, concentrated on pure resins and a combination of Resin—SBS, SIS, APAO, and Polyamide—with C5 as a base to characterize melting behavior, viscosity, and mechanical attributes at relatively low additive contents (up to 10%). These small-proportion blends were used to assess basic flow, film integrity, and solubility issues. Insights gained, which will be discussed in Section 3.1.1 under Results and Discussion, guided the selection of additives for further optimization.

Building on the initial benchmarking results, the study proceeded with targeted blending of C5 resin and each selected modifier in varying combinations to refine key properties such as viscosity, adhesion, and whiteness. The experimental approach started with C5 and APAO (Table 5) evaluating their performance in combination with varying ratios of calcium carbonate and titanium dioxide to assess melting behavior and preliminary mechanical properties. Subsequently, C5 and SBS formulations (Table 6) were explored to examine the solubility and melting characteristics of SBS, especially when used at higher proportions.

As early SBS tests revealed viscosity challenges, additional steps were taken to incorporate PE wax and a medium-temperature modifier (Table 7 and Table 8). This systematic variation in resin-to-additive ratios allowed the balance of brightness, mechanical strength, and crack resistance across a spectrum of C5–SBS blends. Recognizing that SBS encountered fusion difficulties at elevated loadings, the study then turned to SISas a partial or full substitute (Table 9 and Table 10). By leveraging SIS’s lower melting complexity, these co-modification experiments sought to improve ductility without introducing excessive surface stickiness.

In parallel, Polyamide was investigated to address ongoing flexibility concerns (Table 11 and Table 12). Its higher softening point and reinforcement potential offered a distinct route to enhancing the mechanical robustness of the blend. To mitigate phase-separation issues observed with wax-containing batches, one set of experiments removed PE wax (Table 11), while another introduced SIS into the C5–Polyamide matrix (Table 12) with the aim of further improving elongation.

These iterative blending trials systematically adjusted the ratios of resin, powder additives, and glass beads to refine the material properties essential for effective road markings. The process culminated in identifying an optimized thermoplastic formulation, which was subsequently processed into 2 mm-thick sheets for prototyping which are used for field validation.

#### 2.2.3. Validation Test

Following the optimization of the road marking material, thermoplastic road marking materials were tested in accordance with the KS M 6080: Road Marking Paint standard [25] to evaluate their suitability under both laboratory and real-world conditions. Test specimens were prepared by heating the material to 180–220 °C and applying it onto ethanol-cleaned steel plates (150 mm × 150 mm, 2 mm thick) to a thickness of 2 mm ± 0.1 mm. The plates were cooled at 20 °C ± 2 °C for 24 h before testing.

To ensure thorough validation, the following key performance tests were conducted on the thermoplastic materials, all adhering to the KS M 6080:

1. Abrasion Resistance: A Taber Abrasion Tester (Model 5135) with CS-17 wheels under a 1 kg load for 100 cycles was used to evaluate resistance to wear under mechanical stress.
2. Brightness: Measurements were performed using a FLIR E50 retro reflectometer, (FLIR Systems, Seoul, South Korea) at a 2.29° observation angle under standardized lighting conditions to assess visibility performance.
3. Water Resistance: Specimens were submerged in distilled water at 25 °C ± 1 °C for 24 h, followed by visual and magnified inspections to identify any defects, such as cracks or discoloration.
4. Heavy Metal Content: Lead and cadmium levels were analyzed via ICP-MS following nitric acid-peroxide digestion, ensuring compliance with the KS M 6080 standard limits for environmental and health safety.

Tests were conducted under standardized laboratory conditions (23 °C ± 2 °C, 50% ± 5% relative humidity).

Field validation trials were conducted at two locations to evaluate the performance of the thermoplastic material under controlled and real-world conditions. At the Chuncheon test site, the Korea Construction and Living Environment Testing Institute, thermoplastic markings were applied to specimens representing newly paved and existing pavement surfaces. The markings were engraved and inlaid into asphalt and concrete pavements, with heating used to create indentations for embedding the material. After placement, the markings were reheated to ensure full integration with the pavement. These trials focused on assessing material behavior, adaptability, and consistency under standardized conditions. At Kangwon National University, full-scale trials were performed on the entrance and exit roads of the main stadium to evaluate the material under actual traffic and environmental conditions. Both slip resistance and adhesive strength were assessed using standardized methods.

Skid resistance testing followed the KS F 2375:2016 [26] standard and utilized a Skid Resistance Tester Model 48-PV0190 (CONTROLS Group, Milan, Italy). Polymer-modified asphalt and concrete surfaces were prepared to standard dimensions, wetted to simulate wet road conditions, and tested to measure the British Pendulum Number (BPN). The results quantified the slip resistance of the thermoplastic material across different pavement types.

Adhesive strength testing adhered to the KS F 2386:2013 standard [27]. Tensile forces were applied directly to the bonded layers until failure, using a Proceq DY-225, pull-off tester (Screening Eagle Technologies S.A., Schwerzenbach, Switzerland)at a loading rate of 0.1 MPa/s. The maximum tensile load (P) and bonded area (A) were recorded, and adhesive strength (S = P/A) was calculated to quantify bonding performance. Specimens were tested on-site without the need for core sample extraction.

## 3. Results and Discussion

### 3.1. Development and Optimization of Thermoplastic Road Marking Material

#### 3.1.1. Benchmarking Phase

**Figure 5:**
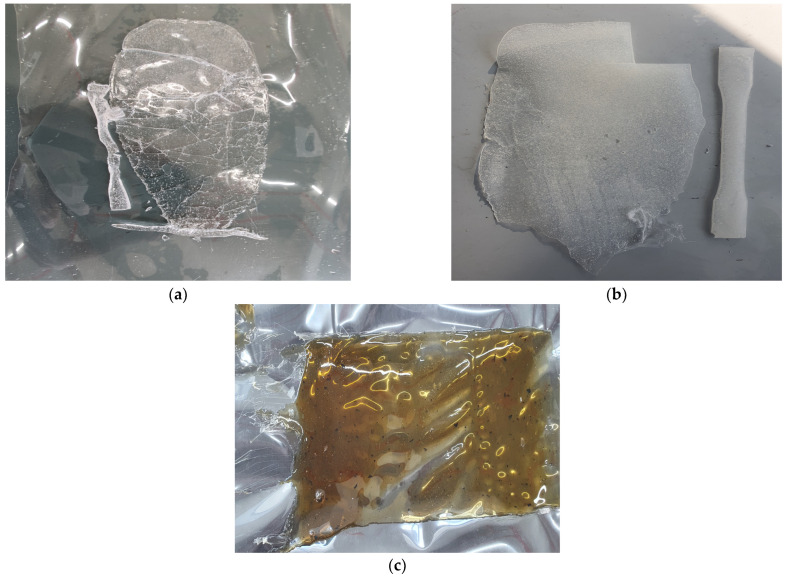
Melting behavior of pure resins: (**a**) C5 resin; (**b**) APAO resin; (**c**) polyamide resin.

The evaluation of pure resins yielded insights into their intrinsic properties and practical viability. The findings reveal that C5 resin, while characterized by its low viscosity and rapid melting rate, possesses an inherent brittleness and a complete absence of film ductility, as illustrated in Figure 5a. These attributes render it unsuitable for standalone applications due to its structural rigidity and pronounced susceptibility to fracture. Although C5’s processability is enhanced by favorable melting behavior, its mechanical deficiencies necessitate strategic modification for practical deployment.

In contrast, APAO and Polyamide resins demonstrated markedly superior performance, exhibiting both low viscosity and commendable film ductility (Figure 5b,c). These properties indicate their significant potential as modifiers capable of imparting the requisite flexibility and resilience to otherwise brittle C5 formulations. The ability of APAO and Polyamide to introduce pliancy without compromising processing efficiency positions them as viable candidates for enhancing the mechanical profile of C5-based composites. Nonetheless, their distinct rheological behaviors require meticulous formulation strategies to achieve an optimal balance between flexibility and structural integrity.

Following the evaluation of pure resins, the study focused on benchmarking blends of C5 resin with small proportions of modifiers (up to 10% modifier content), as described in Section 2.2.2 of the Methods. This preliminary stage established a baseline for each modifier’s impact on viscosity, flexibility, and mechanical stability when combined with C5. Table 12 presents some of the key observations from these early tests, which guided subsequent, more targeted experiments.

**Figure 6:**
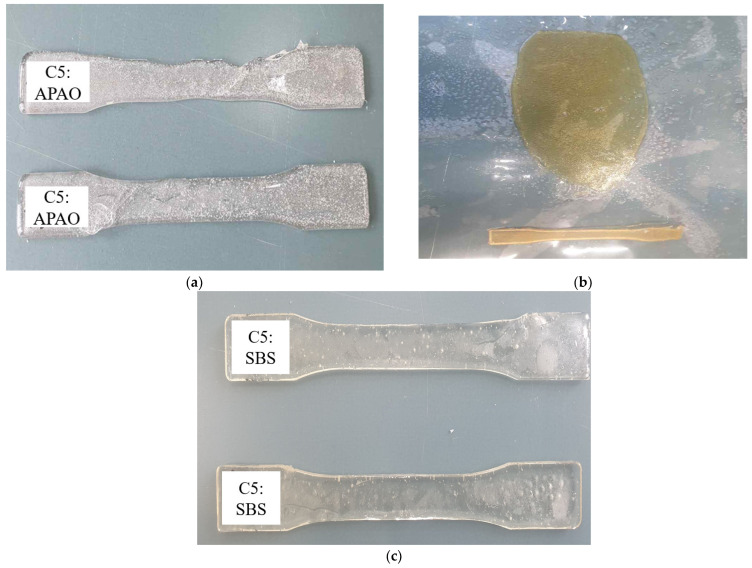
Performance of resin combinations (90:10): (**a**) C5: APAO—improved viscosity; (**b**) C5: Polyamide—reduced viscosity, limited flexibility; (**c**) C5:SBS—poor solubility.

**Figure 7:**
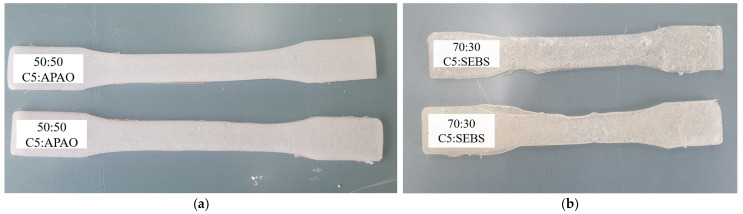
Performance of resin combinations: (**a**) C5: APAO (50%:50%)—material sagging; (**b**) C5: SEBS = 70%:30%—poor melting.

Figure 6 and Figure 7 illustrate the baseline and higher-ratio modifier blends, respectively. In Figure 5, the 90:10 ratios of C5 to each modifier highlight improved viscosity and flow characteristics with APAO or Polyamide but limited flexibility at that proportion, as well as poor solubility in the C5:SBS blend, aligning with the general observations in Varga and Bárány [22] and Yu et al. [21] that highlight the importance of balancing flow properties against elasticity. In contrast, Figure 6 captures the extreme cases of 50:50 C5:APAO (exhibiting high flexibility but structural sagging) and 70:30 C5:SEBS (showing inadequate melting and elevated viscosity).

Utilizing a 90:10 C5:Modifier ratio served as a practical baseline to assess the dominant effects of each modifier. At this composition, both APAO (Figure 6a) and Polyamide (Figure 5b) effectively reduced viscosity and enhanced flow characteristics. However, neither additive provided the requisite flexibility for road-marking applications when used at this loading. In contrast, SBS at the same 90:10 ratio (Figure 6c) demonstrated even less promise, exhibiting incomplete melting and poor solubility within the C5 matrix—a phenomenon also noted by Hemmati et al. [20] for SBS-rich asphalt binders. Summary of performance observations are tabulated in Table 13.

At high loadings, APAO (50:50) significantly increased film ductility, albeit to the point of sagging as shown in (Figure 7a). This observation corroborates the mechanical property shifts reported by Varga and Bárány [22] for APAO-based single-polymer composites. Meanwhile, SEBS at a 70:30 ratio produced elevated viscosity and melting issues akin to those described in Ji et al. [15] for thermoplastic-polyimide mixtures. These trials, therefore, clarified that while small amounts of APAO or Polyamide help reduce viscosity, higher loadings jeopardize structural integrity.

Overall, these observations delineated the processing limitations associated with each modifier. Lower concentrations of APAO or Polyamide were advantageous for viscosity reduction but insufficient alone to achieve the desired flexibility. Meanwhile, SBS and SEBS exhibited notable solubility and melting issues at the tested ratios. This initial stage clarified the dominant effects of each modifier, informing the more detailed formulation optimization described in Section 2.2.2 (Table 4, Table 5, Table 6, Table 7, Table 8, Table 9, Table 10 and Table 11).

#### 3.1.2. Optimization Phase

C5 and APAO Resin Mixing Experiment: The initial benchmarking indicated that APAO and Polyamide produced broadly comparable effects on viscosity and flow properties. Subsequent experiments, therefore, explored a range of C5-to-APAO ratios and evaluated the influence of different powder additives on melting performance, viscosity, durability, and visual clarity. Table 5 summarizes the tested formulations in this series. When CaCO_3_ served as the sole powder additive, the mixture appeared unacceptably dull, hindering visibility for road markings. Conversely, relying exclusively on TiO_2_ yielded an overly bright whiteness that reduced discernibility. In contrast, APAO alone offered efficient melting, suitable viscosity, and smooth flow characteristics. However, re-melting trials revealed soot formation, suggesting thermal instability under typical on-site heating conditions. Consequently, identifying a balanced approach that harnesses APAO’s favorable melting properties while mitigating instability became a key target for the subsequent refinement steps.

C5 and SBS Resin Mixing Experiments: Building on the preliminary verification in Section 2.2.2, SBS was further investigated to determine whether its inherent flexibility could be harnessed despite its high melting point and viscosity. Trials using ratios of 100% SBS, 90:10, and 66.7:33.3 (Table 6) confirmed that the pure SBS mix suffered from incomplete melting and poor heat conduction, while lower proportions yielded insufficient flexibility or elevated viscosity. Attempts to refine this behavior led to the inclusion of PE wax and a medium-temperature modifier (Table 7) to reduce viscosity and melting temperature. Although these additives partially improved flow, incorporating CaCO_3_, TiO_2_, and glass beads introduced new trade-offs. In particular, CaCO_3_ alone produced inadequate whiteness, whereas mixing CaCO_3_ with TiO_2_ improved brightness but caused intermittent flowability problems and inconsistent coverage. Excessive powder content also lowered the cured film’s flexibility, resulting in visible cracking. Varying the C5:SBS ratio (Table 8) revealed that increasing resin content generally facilitated melting and flow, but higher powder content constrained ductility. Collectively, these findings demonstrated that SBS, when used as the primary modifier, imposes significant limitations on melting stability and mechanical adaptability, prompting the need to explore alternative or complementary modifiers.

C5 and SIS Resin Mixing Experiment: In response to the difficulties noted with SBS, SIS was introduced as an alternate or co-modifier (Table 9 and Table 10). SIS offered more manageable melt characteristics, but balancing ductility with overall rigidity remained challenging. Partial replacement of SBS with SI (maintaining the overall 60:40 C5:Modifier ratio) showed modest gains in film flexibility compared to prior C5–SBS blends, yet excessive SIS (above approximately 40%) substantially weakened the cured film. Conversely, lower SIS loadings (~20%) proved insufficient for achieving the desired flexibility. Similar observations appear in polymeric sulfur-modified asphalt studies, where maintaining a balance between ductility and stiffness is crucial [17]. Surface contamination emerged under certain ratios, suggesting SIS, while beneficial for certain properties, does not wholly remedy performance gaps when used as a sole or dominant modifier.

C5 and Polyamide Resin Mixing Experiment: The next stage of optimization tested Polyamide as a primary co-resin (Table 11 and Table 12), owing to its lower melting viscosity and higher softening point than SBS or SIS. Increasing Polyamide content improved the thermoplastic’s mechanical stability and formability, although flexibility remained marginal. Including PE wax caused unwanted phase separation, leading to its exclusion in subsequent trials. While the resulting C5–Polyamide blends showed promising shear strength and thickness uniformity, their flexibility still fell short of targeted performance. However, flexibility remained suboptimal, and attempts to include SIS for extra ductility led to surface stickiness, echoing the potential for interface issues described in references [16,17,18,19]. Although Polyamide significantly bolstered viscosity control and overall structural integrity, achieving superior flexibility required more precise modifications to resin–powder interactions.

#### 3.1.3. Final Selection

**Figure 8:**
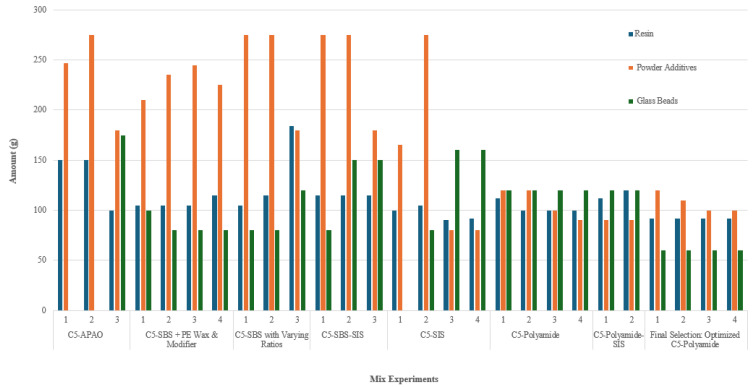
Distribution of resin, powder additives, and glass beads across all experiments.

Drawing on the previous iterative experiments, a final series of blends (Table 14) adjusted the resin-to-powder ratio below 1:1.1 to avoid soot formation during re-melting and to stabilize thermal behavior. Increased TiO_2_ content, coupled with moderated CaCO_3_ and glass bead additions, yielded high whiteness and adequate retroreflectivity without compromising mechanical strength. This composition delivered adequate retroreflectivity without compromising structural integrity, consistent with the optimum visibility–durability balance that Owusu et al. [6] and Zhao et al. [23] emphasize for long-term pavement markings. Mixture 4 of Table 13 emerged as the most effective candidate for road marking applications, offering robust thermal stability, visual clarity, and feasible processing characteristics. Figure 8 illustrates how resin, powder, and bead percentages evolved across all formulations, showcasing the systematic progression from limited single-modifier trials to a refined composition capable of meeting durability and visibility demands.

### 3.2. Performance Validation in Laboratory and Field Settings

This study’s validation process consisted of two main stages: indoor laboratory testing and outdoor field testing. The indoor testing focused on evaluating the optimized thermoplastic material under controlled conditions, while the field trials assessed its performance in real-world applications across two locations: the Chuncheon site of the Korea Construction & Living Environment Test Research Institute for pre-tests, and the entrance and exit roads of the main stadium at Kangwon National University in Chuncheon, South Korea for full-scale application.

#### 3.2.1. Laboratory Testing

**Figure 9:**
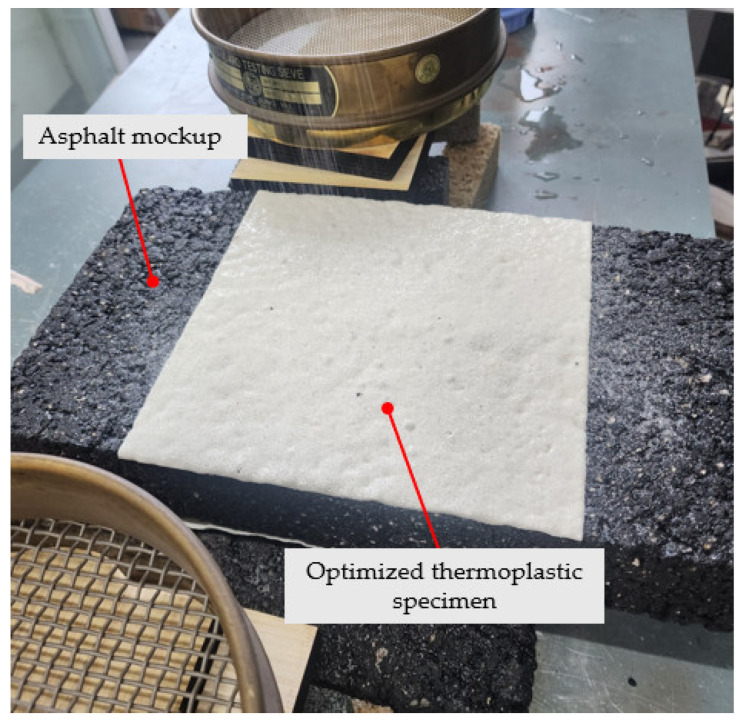
Thermoplastic test specimen evaluated according to KS M 6080 standard for durability and safety compliance.

The primary objective of this study was to develop and validate an optimized thermoplastic road marking system. Consequently, performance metrics were assessed exclusively on the final optimized mix to confirm its suitability for practical applications. Key performance indicators, including abrasion resistance, brightness, water resistance, and heavy metal content, were evaluated. Figure 9 illustrates a thermoplastic test specimen evaluated in accordance with the KS M 6080 standard.

The results demonstrated that the material met or exceeded all target performance indicators, as shown in Table 15. The systematic blending of thermoplastic resins and the optimization of inlay patterns and heating techniques contributed to a product that performs well under various conditions. The material’s durability, as indicated by abrasion resistance tests, ensures it can withstand the rigors of high-traffic areas. The achieved brightness and water resistance further enhance its suitability for safety-critical applications. Compliance with lead and cadmium content standards ensures the material is environmentally and health safe.

#### 3.2.2. Field Trials and Inlay Application

**Figure 10:**
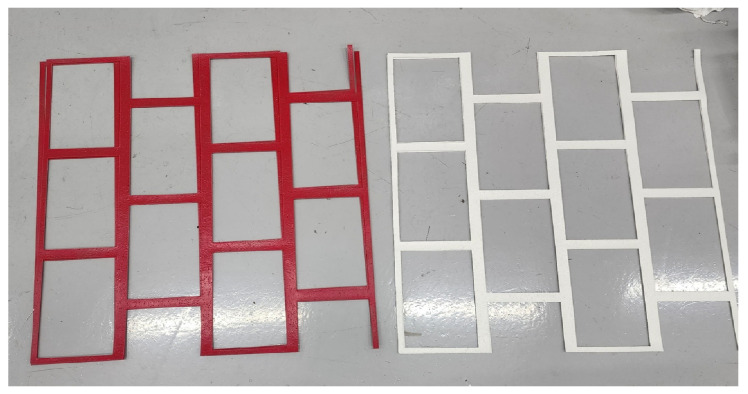
Final prototype of the thermoplastic road marking system.

**Figure 11:**
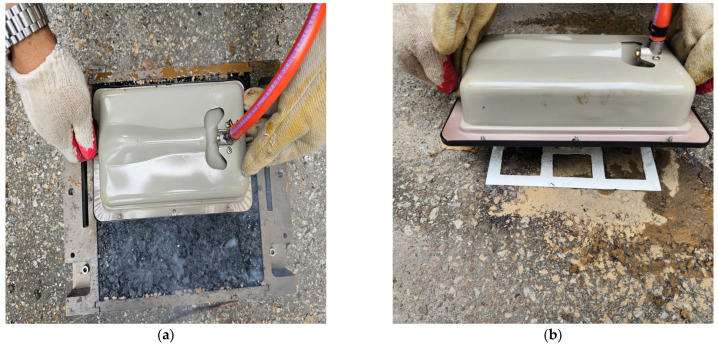
Pre-testing results for thermoplastic road markings on asphalt. (**a**) Marking applied to a newly paved surface; (**b**) Marking applied to an existing pavement surface.

The prototype of the developed thermoplastic material, depicted in Figure 10, was tested in two phases. Pre-tests at the Korea Construction & Living Environment Test Research Institute provided a controlled setting to refine engraving and inlaying on both fresh and aged asphalt specimens (Figure 11). Full-scale application followed at Kangwon National University’s main stadium roads, exposing the markings to active traffic conditions.

The first pre-test took place on the grounds adjacent to the Korea Construction & Living Environment Test Research Institute, located in Chuncheon, Gangwon Province, South Korea. This site was selected due to its controlled environment and accessibility for conducting initial evaluations. A two-phase heating approach was deployed to (1) preheat and engrave the asphalt, then (2) embed the thermoplastic sheets. This process ensures mechanical interlock, effectively reducing delamination [16,18,19]. As shown in Figure 11a, the process was tested on specimens representing newly paved surfaces, while Figure 11b illustrates the process on existing pavement conditions.

Following the pre-tests, full-scale field trials were conducted on the entrance and exit roads of the main stadium at Kangwon National University in Chuncheon, South Korea. This site, selected in collaboration with the university’s facilities department, offered active traffic conditions that provided a realistic assessment of the material’s performance. The developed heating system preheated the asphalt surface, engraved the desired patterns, and seamlessly integrated the thermoplastic sheets. The same two-phase heating process was employed.

**Figure 12:**
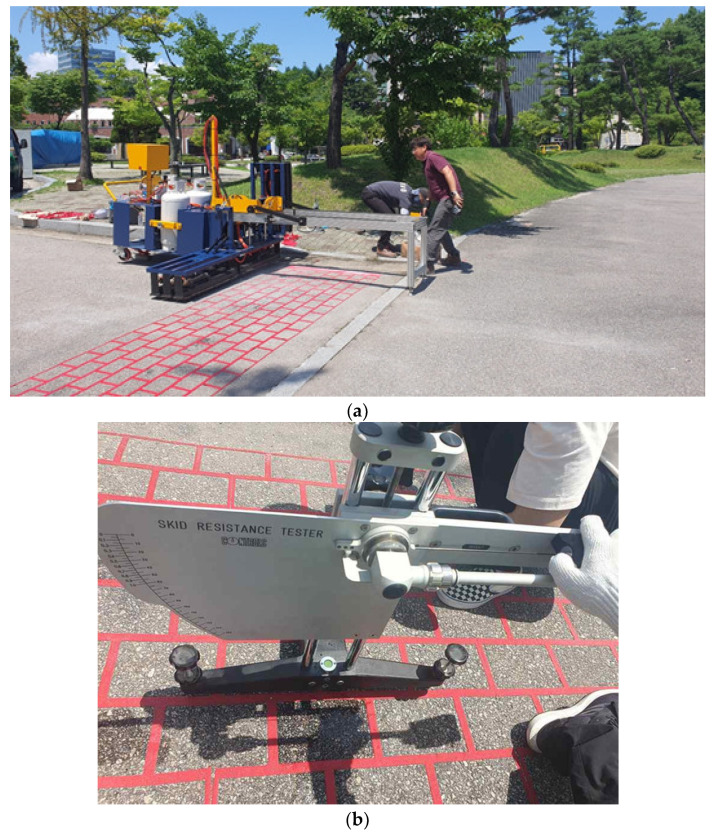
Field Testing: (**a**) On-site deployment; (**b**) Skid resistance test conditions.

Figure 12 illustrates key aspects of the system. Figure 12a shows the on-site deployment, highlighting the modular and adaptable design for large-scale applications. The engraving process securely embedded the thermoplastic sheets into the grooves, enhancing structural integrity and minimizing delamination risks. Skid-resistance tests (Figure 12b) confirmed stable friction, even under wet conditions, paralleling the improved adhesion performance reported by references [17,18] for rough asphalt surfaces. These results validate the heating and bonding methodology as a reliable, scalable solution for modern road infrastructure.

The field trials demonstrated that the thermoplastic road markings remained intact, visually clear, and durable after application. This was achieved through precise heating and bonding processes that ensured uniform integration of the markings with the asphalt. As shown, the heating system operated effectively, producing well-defined and long-lasting road markings that withstood real-world traffic stresses. The results of the full-scale trials are summarized in Table 16.

### 3.3. Results Synthesis

The performance metrics obtained from this study carry significant implications for the deployment and effectiveness of thermoplastic road markings. Compared to traditional cold-applied paints or existing pre-formed thermoplastic sheets, the proposed system offers substantial advantages in durability, visibility, and bonding performance. Traditional markings often suffer from premature wear, delamination, and reduced visibility under high-traffic conditions. In contrast, the developed system employs a precise two-phase heating process that enhances bonding between the thermoplastic material and the pavement, resulting in increased durability and reduced maintenance costs.

Laboratory tests revealed mass loss below 500 mg after 100 rotations, indicating high durability under mechanical wear and traffic conditions. This aligns with the durability requirements highlighted by Owusu et al. [6], ensuring markings withstand continuous friction and pressure from vehicular traffic. Achieving a brightness level of ≥0.5 ensures high visibility under various lighting conditions, crucial for road safety. Enhanced brightness minimizes accident risks by providing clear visual cues, particularly during nighttime or adverse weather, as emphasized by Zhao et al. [28]. The absence of cracks, swelling, wrinkles, or discoloration after 24-h submersion confirms strong resistance to moisture and environmental exposure. This strong water resistance ensures markings remain intact and functional in wet conditions, preventing safety hazards caused by degraded markings, as discussed by Jo et al. [16]. Adhesive strength tests demonstrated values ranging from 1.0 to 1.1 MPa, meeting thresholds for effective road marking applications. This strong bonding performance reduces delamination risks, ensuring markings remain securely attached even under heavy traffic loads. These findings corroborate adhesion mechanisms identified by Varga and Bárány [22] and Wang et al. [19]. Field trials showed consistent skid resistance values up to 70 BPN, enhancing road safety by providing reliable traction, even in wet conditions. This level of skid resistance prevents tire slippage and reduces accident risks, aligning with safety performance metrics outlined by Owusu et al. [6].

The combination of polymeric resins parallels the synergy observed in other studies offering enhanced flow, viscosity control, and mechanical adaptability. This multifaceted approach ensures optimal performance across diverse environmental and traffic conditions. Furthermore, the modular heating system allows efficient deployment over large road segments, with adjustable height and rails ensuring compatibility with varying road geometries. Precise temperature control and uniform heat distribution minimize thermal losses, enhancing operational efficiency. The modular design supports seamless transitions between road segments, making it suitable for large-scale applications and reducing energy consumption compared to traditional methods.

The heating system requires considerable energy input, potentially limiting deployment in remote or power-constrained areas. Additionally, long-term studies under extreme temperature swings or heavy traffic loads are necessary to validate geographic suitability [19]. Variations in traffic patterns and environmental conditions may also impact long-term durability and should be addressed in future research.

By exceeding benchmarks for abrasion resistance, brightness, and skid resistance, thermoplastic markings significantly enhance road safety, reducing accidents caused by poor visibility or slippery surfaces. Increased durability and reduced maintenance translate to lower lifecycle costs for road authorities, allowing more efficient allocation of resources. Compliance with lead and cadmium content standards ensures environmental and health safety. Minimizing VOC emissions and enhancing material longevity reduce the environmental footprint of road marking applications. The two-phase heating process reduces labor intensity and improves worker safety, lowering installation costs and accelerating project timelines.

Future work should explore renewable energy sources for on-site heating, test advanced polymer blends for colder climates, and conduct multi-year performance monitoring to substantiate longevity and performance claims under real-world conditions. Investigating alternative formulations to enhance flexibility under extreme temperatures and optimizing filler interactions will further refine the system’s applicability across diverse environments.

## 4. Conclusions

This study introduces a novel pre-formed thermoplastic road marking system that integrates an optimized resin formulation with a two-phase heating process, thereby enhancing both the durability and visibility of road markings while streamlining the application procedure. The primary contributions of this research:

- Optimized Resin Formulation: Developed a unique thermoplastic blend including C5 petroleum resin, SBS resin, polyamide resin, wax, plasticizers, calcium carbonate, titanium dioxide, and glass beads, significantly improving durability, flexibility, and visibility.
- Two-Phase Heating Process: Pioneered a method that involves engraving the asphalt surface followed by the application of pre-formed thermoplastic sheets, ensuring consistent adhesion and minimizing application errors.
- Advanced Heating System: Utilized rectangular ceramic heaters with precise temperature control to achieve uniform heating of both asphalt surfaces and thermoplastic sheets, enhancing application efficiency and safety.
- Comprehensive Field Validation: Demonstrated the system’s reliability and performance on both newly paved and existing asphalt surfaces, showcasing strong adhesion, high durability, and excellent visibility in real-world conditions.

Compared to traditional road marking techniques, this integrated approach offers significant improvements in application efficiency, safety, and overall performance. The system’s adaptability allows for its implementation across various scenarios, including bike lanes, bus lanes, and safety zones, contributing to more resilient and long-lasting road infrastructure.

However, the study is limited by the need for further evaluation of the system’s performance under extreme climatic conditions and prolonged heavy traffic loads. Future research should address these limitations to enhance the technology’s robustness and expand its applicability to diverse global infrastructure environments. Additionally, exploring renewable energy sources for on-site heating and optimizing polymer blends for colder climates will further refine the system’s effectiveness.

In conclusion, the proposed road marking system represents a step in advancing road safety and infrastructure durability. By synthesizing diverse polymeric modifiers, controlling filler levels, and mitigating safety hazards, this system demonstrates the substantial potential for modern, large-scale infrastructure projects worldwide.

## Figures and Tables

**Table 1:** Advantages and limitations of existing road marking technologies.

Road Marking Mechanism	Advantages	Limitation
Heat-Weldable Tapes	- Excellent visibility - Variety of colors - Superior design options	- High cost - Specialized installation - Lifespan of ~12 months
Pre-formed Thermoplastic Markings	- Customization - Durability - Enhanced retroreflectivity	- High cost - Complex installation - Safety risks (e.g., fire hazards) - Frequent maintenance
Paint-Based Coatings	- Lower cost - Easy to apply	- Rapid wear - Environmental degradation - High maintenance
MMA Resins	- Durable - Resistant to wear - Fast curing	- Hazardous chemicals - Complex application
Adhesive-Based Markings	- Capable of digital printing - Excellent visibility - Easy installation and removal	- High cost - Short lifespan

**Table 2:** Summary of key studies.

Authors	Methods	Results	Limitations	Research Gap
Ji et al. [15]	Differential Scanning Calorimetry (DSC), Dynamic Mechanical Analysis (DMA)	Improved flexibility, tensile strength, reduced melting temperature	Limited to specific modifiers; no long-term impact analysis	Explore broader polymeric modifiers and long-term effects
Jo et al. [16]	Three-point bend testing, Single edge notch bend testing, Shear adhesion testing	Better adhesion with lower modulus PPMs	Limited to two commercial PPMs	Investigate diverse PPMs and adhesion mechanisms
Kavussi et al. [17]	Fatigue, freeze-thaw tests, Response Surface Method (RSM)	Crumb rubber improved fatigue life by up to 70%; polymeric sulfur reduced ductility and flexibility	Did not fully explore underlying adhesion mechanisms between modified mixtures and asphalt substrates	Understand the interplay between mechanical interlock and chemical bonding in adhesion performance
Zheng and Zheng [18]	Adhesion work analysis, asphalt compatibility tests	Optimal adhesion with styrene-ethylene/butylene-styrene asphalt	Focused only on asphalt and aggregates	Assess polymeric modifiers’ impact on road marking adhesion
Wang et al. [19]	Molecular Dynamics (MD) simulations, contact angle analysis	Short-term aging enhanced interfacial adhesion	Focused on short-term aging effects	Evaluate long-term adhesion performance
Hemmati et al. [20]	Superpave testing method, Rotational Viscometer (RV), Dynamic Shear Rheometer (DSR), Bending Beam Rheometer (BBR), Aging protocols (RTFO and PAV)	SBS improved flexibility, co-modification enhanced viscosity and rutting resistance	Focused on SBS binders only	Explore alternative polymers like SIS and APAO
Yan et al. [21]	Superpave binder grading system, Rolling Thin Film Oven (RTFO) test	Alternative polymers comparable to SBS in moisture and rutting resistance	No climate condition variability tested	Assess performance under diverse environments
Varga and Barany [22]	Film-stacking, tensile and impact tests, Scanning Electron Microscopy (SEM)	APAO enhanced consolidation and adhesion	Conducted in lab settings only	Evaluate long-term durability in real-world conditions
Owusu et al. [6]	Empirical model review, retroreflectivity analysis	Identified factors leading to retroreflectivity loss	Did not assess pigment and bead effects	Investigate pigment and bead impacts on durability
Zhao et al. [23]	Data analysis, field surveys, MLR, LightGBM	Accurate retroreflectivity prediction (R² = 0.942)	Limited to one region, no material analysis	Optimize pigment and bead concentrations for contrast

**Table 3:** Resin material properties.

Material	Product	Properties	Purpose
Resin	C5	Softening Point: 103 °C Melt Viscosity: 200 cps at 180 °C Aromatic Content: <1%	Primary binder
Resin	Polyamide Resin	Melting Point: 135 ± 5 °C, Viscosity: 4500 ± 1000 cps (200 °C), Shore A Hardness: 70	Reinforcement
Plasticizers	APAO	Melting Point: 100–140 °C Viscosity: >200 cP at 135 °C Excellent Oxidation Resistance	Controls viscosity and improves flow
Lubricant	PE Wax	Melting Point: 80–115 °C Viscosity: >10 cP (135 °C) Molecular Weight: 600–1300 Daltons	Surface treatment, improves flow properties

**Table 4:** Powder materials properties.

Material	Product	Properties	Purpose
Filler	Heavy Calcium Carbonate (CaCO_3_)	Particle Size: 15–20 μm Density: ~2.7 g/cm^3^ Whiteness: High	Filler for bulk and stability
Inorganic Pigment	Titanium Dioxide (TiO_2_)	Opacity: High UV Resistance: Excellent Particle Size: 0.2–0.3 μm	Whiteness
Inorganic Pigment	Iron Oxide	Color Stability: High Weather Resistance: Excellent	Color Development
Reflective Material	Glass Beads	Refractive Index: ~1.5 Transparency: High Retroreflection: 250–300 mcd/lux.m^2^	Improves retroreflectivity for visibility

**Table 5:** Formulation ratios of C5 and APAO with additives.

Category		Mix 1 (g)	Mix 2 (g)	Mix 3 (g)
Resin	C5	90	90	60
	APAO	60	60	40
Powder	CaCO_3_	247	-	165
	TiO_2_	-	360	75
Glass bead		-	-	175
Total		397	510	515

**Table 6:** Performance results of C5 and SBS thermoplastic formulations.

Material	Ratio	Observation
SBS	100%	Poor surface melting and mixing. Low thermal conductivity caused uneven heating and burning.
C5: SBS	90%: 10%	Low viscosity facilitated smooth flow, but flexibility was insufficient, and SBS did not fully melt.
C5: SBS	66.7%: 33.3%	Excessively high viscosity hindered molding and handling, resulting in poor workability and film formation.

**Table 7:** C5 and SBS formulations with PE wax and temperature modifier.

Category		Mix 1 (g)	Mix 2 (g)	Mix 3 (g)	Mix 4 (g)
Resin	C5	60	60	60	60
	SBS	30	30	30	40
	PE Wax	12	12	12	12
	Medium-Temperature Modifier	3	3	3	3
Powder	CaCO_3_	210	150	200	150
	TiO_2_	0	85	45	75
Glass bead			80	80	80
Total		415	420	430	420

**Table 8:** Varying ratios of C5 and SBS formulations with additives.

Category		Mix 1 (g)	Mix 2 (g)	Mix 3 (g)
Resin	C5	60	60	80
	SBS	30	40	80
	PE Wax	12	12	24
	Medium-Temperature Modifier	3	3	-
Powder	CaCO_3_	200	200	60
	TiO_2_	75	75	120
Glass bead		80	80	120
Total		415	470	364

**Table 9:** Formulation ratios of C5, SBS, and SIS with additives.

Category		Mix 1 (g)	Mix 2 (g)	Mix 3 (g)
Resin	C5	60	60	60
	SBS	28	32	32
	SIS	12	8	8
	PE Wax	12	12	12
	Medium-Temperature Modifier	3	3	3
Powder	CaCO_3_	200	200	200
	TiO_2_	75	75	75
Glass bead		80	150	150
Total		470	540	585

**Table 10:** Formulation ratios of C5 and SIS with additives.

Category		Mix 1 (g)	Mix 2 (g)	Mix 3 (g)	Mix 4 (g)
Resin	C5	60	60	60	60
	SIS	40	18	21	20
	LDPE	-	12	9	12
	PE Wax	-	12	-	-
	Medium-Temperature Modifier	-	3	-	-
Powder	CaCO_3_	165	200	-	-
	TiO_2_	-	75	80	80
Glass bead		-	80	160	160
Total		265	460	330	332

**Table 11:** Formulation ratios of C5 and polyamide with additives.

Category		Mix 1 (g)	Mix 2 (g)	Mix 3 (g)	Mix 4 (g)
Resin	C5	60	60	60	60
	Polyamide	40	40	40	40
	PE Wax	12	0	0	0
Powder	CaCO_3_	60	60	40	30
	TiO_2_	60	60	60	60
Glass bead		120	120	120	120
Total		352	340	320	310

**Table 12:** Formulation ratios of C5, polyamide, and SIS with additives.

Category		Mix 1 (g)	Mix 2 (g)
Resin	C5	60	60
	Polyamide	40	40
	SIS	12	20
Powder	CaCO_3_	30	30
	TiO_2_	60	60
Glass bead		120	120
Total		322	330

**Table 13:** Performance observations of thermoplastic formulations.

Modifier	Blend Ratio	Viscosity	Flexibility	Solubility	Additional Observations
APAO	90:10 C5	Improved	Limited	Poor	Enhanced flow characteristics
Polyamide	90:10 C5	Improved	Limited	Poor	Enhanced flow characteristics
SBS	90:10 C5	N/A	N/A	Poor	Poor solubility in C5:SBS blend
APAO	50:50 C5	High	High	N/A	Structural sagging observed
SEBS	70:30 C5	Elevated	Inadequate	N/A	Inadequate melting and elevated viscosity

**Table 14:** Optimal thermoplastic formulation for road marking applications.

Category		Mix 1 (g)	Mix 2 (g)	Mix 3 (g)	Optimal Mix
Resin	C5	40	40	40	40
	Polyamide	52	52	52	52
Powder	CaCO_3_	60	55	50	40
	TiO_2_	60	55	50	60
Glass bead		60	60	60	60
Total		272	262	255	272

**Table 15:** Target performers’ indicators tested according to KS M 6080.

Performance Metric	Result	Implication
Abrasion Resistance	Mass loss < 500 mg after 100 rotations	Indicates high durability under mechanical wear and traffic conditions.
Brightness	Brightness ≥ 0.5	Ensures high visibility under various lighting conditions, crucial for road safety.
Water Resistance	No cracks, swelling, wrinkles, or discoloration after 24 h submersion	Confirms strong resistance to moisture and environmental exposure.
Lead Content	≤0.06	Adheres to safety and environmental standards, ensuring minimal health risks.
Cadmium Content	≤0.01	Complies with permissible health and safety regulations for environmental sustainability.

**Table 16:** Field testing results.

Test Items	Unit	Result Value
Skid Resistance (Asphalt)	BPN	70
Skid Resistance (Polymer—Aggregate)	BPN	65
Skid Resistance (Polymer—Entirety)	BPN	50
Adhesive Strength (Asphalt)	MPa	1.0
Adhesive Strength (Asphalt)	MPa	1.1
Adhesive Strength (Asphalt)	MPa	1.1

## Data Availability

The data supporting this study’s findings are available on request from the authors. However, due to confidentiality concerns, the data are not publicly accessible.

## References

[1] Akuh R., Zhong M., Raza A. (2022). Evaluating a Proposed Urban Transportation System Using Advance Transport and Land-Use Modelling Framework. Adv. Sci. Technol. Res. J..

[2] Global Land Transport Infrastructure Requirements—Analysis. https://www.iea.org/reports/global-land-transport-infrastructure-requirements.

[3] Ng C.P., Law T.H., Jakarni F.M., Kulanthayan S. (2019). Road Infrastructure Development and Economic Growth. IOP Conf. Ser. Mater. Sci. Eng..

[4] CDC Global Road Safety. https://www.cdc.gov/transportation-safety/global/index.html.

[5] Safety Improvement Division (2017). Special School Zone Inspections Conducted After Multiple Traffic Accidents.

[6] Owusu V., Tuffour Y.A., Obeng D.A., Salifu M. (2018). Degradation of Retro-Reflectivity of Thermoplastic Pavement Markings: A Review. Open J. Civ. Eng..

[7] Calvo-Poyo F., de Oña J., Garach Morcillo L., Navarro-Moreno J. (2020). Influence of Wider Longitudinal Road Markings on Vehicle Speeds in Two-Lane Rural Highways. Sustainability.

[8] Aqsha A., Winoto H.P., Adhi T.P., Adisasmito S., Ramli Y., Siddiq L., Pratama F.B., Ramdani M.R., Indarto A. (2023). Sequential Esterification—Diels-Alder Reactions for Improving Pine Rosin Durability within Road Marking Paint. Molecules.

[9] Heydari S., Hickford A., McIlroy R., Turner J., Bachani A. (2019). Road Safety in Low-Income Countries: State of Knowledge and Future Directions. Sustainability.

[10] Daedong Safety Co., Ltd. Hot Melt Tape. http://www.daedongsafety.co.kr/english/product/product_03.htm.

[11] Design Marking (Self-Adhesive Signs and Freeform) Green Factory. https://www.greenfactory114.com/page/sub3_2.php.

[12] 3M™ Stamark™ Pavement Marking Tape Symbols & Legends Series 270. https://www.3m.com/3M/en_US/p/d/b00010929/.

[13] PPG Duratherm ENNIS-FLINT^®^ by PPG DURATHERM^®^ Inlaid Surface System. https://www.ppg.com/traffic/en-US/products/preformed-thermoplastic/trafficscapes-8qjxbgn6lp/www.ppg.com/traffic/en-US/products/preformed-thermoplastic/trafficscapes-8qjxbgn6lp/duratherm.

[14] PROMAX Industries Raw Materials. https://www.promaxind.com/products/chemicals/.

[15] Ji Y., Zhang Y., Wang P., Li Y., Sui J. (2021). Mechanical and Thermal Properties of Epoxy Resins Modified by a Novel Thermoplastic-Polyimide. Fibers Polym..

[16] Jo H., Giroux M., Erk K.A., Davis C.S. (2022). Mechanical Testing Methods for Evaluating Thermoplastic Permanent Pavement Markings. Transp. Res. Rec. J. Transp. Res. Board.

[17] Kavussi A., Azarnia M., Ayar P., Pedram M. (2022). The Fatigue Behavior of Polymeric Sulfur-Modified Asphalt Mixtures Subjected to Freeze-Thaw Conditioning. J. Thermoplast. Compos. Mater..

[18] Zheng C.F., Zheng S. (2012). Factors of Adhesion between Asphalt and Mineral Aggregates. Appl. Mech. Mater..

[19] Wang Q., Yu R., Fu G., Chen X., Cai L., Xiao Y., Zhang X., Zhu X. (2022). The Short-Term Aging Effect on the Interface and Surface Wetting Behavior of Modified Asphalt Mixtures. Mater. Res. Express.

[20] Hemmati N., Vigneswaran S., Kim H.H., Lee M.-S., Lee S.-J. (2023). Laboratory Evaluation of Asphalt Binders Containing Styrene-Butadiene-Styrene (SBS) and Processed Oil. Materials.

[21] Yan Y., Chun S., Roque R., Kim S. (2016). Effects of Alternative Polymer Modifications on Cracking Performance of Asphalt Binders and Resultant Mixtures. Constr. Build. Mater..

[22] Varga L.J., Bárány T. (2020). Development of Polypropylene-Based Single-Polymer Composites with Blends of Amorphous Poly-Alpha-Olefin and Random Polypropylene Copolymer. Polymers.

[23] Yu Z., Liang Y., Liang W. (2013). Development and Evaluation of α-Asarone Transdermal Patches Based on Hot-Melt Pressure-Sensitive Adhesives. AAPS PharmSciTech.

[24] Zaldua N., Maiz J., De La Calle A., García-Arrieta S., Elizetxea C., Harismendy I., Tercjak A., Müller A.J. (2019). Nucleation and Crystallization of PA6 Composites Prepared by T-RTM: Effects of Carbon and Glass Fiber Loading. Polymers.

[25] (2022). Road Marking Paint.

[26] (2022). Test Method for Slip Resistance of Road Surface (BPT).

[27] (2022). Test Method for Tensile Bond Strength of Road Pavement Attachment Surface.

[28] Zhao L., Ding H., Sun J., Wu G., Xing H., Wang W., Song J. (2023). Prediction of Service Life of Thermoplastic Road Markings on Expressways. Sustainability.

